# A Deep Learning System for Recognizing and Recovering Contaminated Slider Serial Numbers in Hard Disk Manufacturing Processes

**DOI:** 10.3390/s21186261

**Published:** 2021-09-18

**Authors:** Chousak Chousangsuntorn, Teerawat Tongloy, Santhad Chuwongin, Siridech Boonsang

**Affiliations:** 1 Department of Electrical Engineering, School of Engineering, Faculty of Engineering, King Mongkut’s Institute of Technology Ladkrabang, Bangkok 10520, Thailand; chousak.chousangsuntorn@seagate.com; 2 Center of Industrial Robot and Automation (CiRA), College of Advanced Manufacturing Innovation, King Mongkut’s Institute of Technology Ladkrabang, Bangkok 10520, Thailand; teerawat_tongloy@kkumail.com (T.T.); santhad.ch@kmitl.ac.th (S.C.)

**Keywords:** convolution neural networks, optical character recognition, character classification, hard disk drive

## Abstract

This paper outlines a system for detecting printing errors and misidentifications on hard disk drive sliders, which may contribute to shipping tracking problems and incorrect product delivery to end users. A deep-learning-based technique is proposed for determining the printed identity of a slider serial number from images captured by a digital camera. Our approach starts with image preprocessing methods that deal with differences in lighting and printing positions and then progresses to deep learning character detection based on the You-Only-Look-Once (YOLO) v4 algorithm and finally character classification. For character classification, four convolutional neural networks (CNN) were compared for accuracy and effectiveness: DarkNet-19, EfficientNet-B0, ResNet-50, and DenseNet-201. Experimenting on almost 15,000 photographs yielded accuracy greater than 99% on four CNN networks, proving the feasibility of the proposed technique. The EfficientNet-B0 network outperformed highly qualified human readers with the best recovery rate (98.4%) and fastest inference time (256.91 ms).

## 1. Introduction

**Figure 1:**
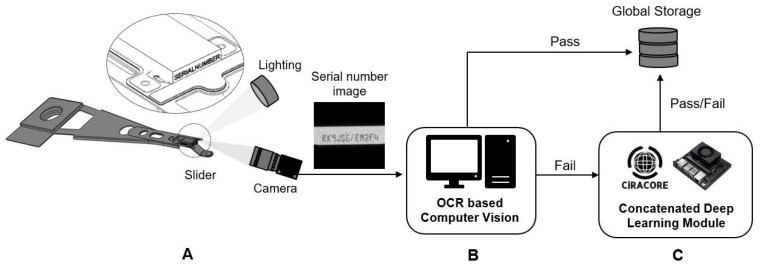
System configuration of the proposed approach for a defect inspection of serial numbers on the slider of HDD. (**A**) OCR image acquisition, (**B**) OCR-based computer vision, and (**C**) OCR-based concatenated deep learning technique.

The head slider is a magnetic head sensor mounted to the tip of a suspension arm, also known as a head gimbal assembly, of a hard disk drive (HDD) that assists in reading and writing components while flying above the hard disk magnetic media surface (Figure 1). A slider was produced using a wafer made of aluminum and titanium. A circular wafer is sectioned into 700-micron pieces and tied to a suspension assembly. Wafer fabrication and mounting, dicing, row chopping, and head parting were all part of the general manufacturing process. During the mechanical slider assembly process, certain defects, such as contamination or scratches, are prevalent. As a result, automatic identification (Auto ID) of sliders is essential for tracking and identifying component process history, processing defects, and product recalls. The Auto ID system employs laser-based serial number printing on individual head sliders. Serial numbers of 12-character length are composed primarily of both numbers (0–9) and letters (A–Z) (A to Z). The serial number is typically captured with a digital camera and saved as an image format (Figure 1A).

In large-scale industrial production, an optical character recognition (OCR) system-based computer vision approach is widely used to read serial numbers on the head slider acquired from more than a hundred slider attachment machines (Figure 1B), with a readable rate of 99.87%. This equates to at least 0.13% of annual production loss due to unidentifiable sliders caused by contamination or scratches on serial numbers during the assembly process. Considering the average cost of product defects, this results in a loss of more than USD 400,000.

Machine learning based on OCR systems is increasingly being introduced in several tasks. Huang et al. suggested a technique for serial number identification of bank cards using a Normalization-Cooperated Gradient Feature (NCGF) and Recurrent Neural Network (RNN) based on Long Short-Term Memory (LSTM), with 90.04% digit string recognition precision [1]. Jang et al. presented character region segmentation and a CNN-based low-quality banknote serial number recognition (SNR) approach, with 99.85% recognition accuracy [2]. Zhu et al. proposed an elevator button recognition framework called OCR-RCNN, which achieved an F1 score of 0.94, 1.00, and 1.00 in the detection task, and an accuracy of 79.6%, 96.5%, and 96.4% in the character recognition task [3]. Sun et al. proposed the SuperOCR system for identifying characters without detecting the location of each character, which was implemented in license plate recognition and watermeter character recognition tasks, respectively, with overall accuracies of 98.7% and 98.0% [4]. Laroca et al. proposed automatic counting (100% accuracy) and identification (recognition rate 99.7%) of train wagons using OCR and deep learning [5]. Kazmi et al. used OCR-based deep learning for tire code detection and text recognition of a moving vehicle using roadside cameras, with a mean accuracy of 86% [6].

Caldeira et al. proposed a convolutional neural network (CNN) classification based on an OCR architecture for implementations of machine learning based on OCR systems in auto ID systems to identify printing ID errors and misidentifications on steel coils [7]. Their procedure, which was designed to work with variations in lighting and printing, provided lower contrast and darker/brighter photographs with greater than 98% precision. Cakic et al. employed the Tesseract OCR engine to recognize serial numbers from wine labels in tracking and tracing individual wine bottles, with an accuracy of 87.5% [8]. Gang et al. demonstrated deep-learning-based coresets for printed circuit board character recognition with 94.7% [9]. Li et al. introduced a CNN framework for automated serial number inspection of ceramic membranes, with an F-score of 95.6% and a precision of 96.5%. Kwon, Hyun, et al. proposed utilizing a deep learning approach for adversarial security by integrating several softmax thresholds on multiple classifiers rather than a single classifier [10]. Nonetheless, there has been no existing literature using OCR-based machine learning in serial number recognition on HDD head sliders [11].

In this article, we propose a novel concatenated method for recognizing serial numbers on HDD head sliders in an industrial production context (Figure 1C). We propose to use the proposed technology to improve the shortcomings of traditional OCR-based computer vision systems. We employ two-state learning techniques, which combine an object detection model based on YOLOv4 with one of four classification models, namely DarkNet-19 [12], EfficientNet-B0 [13], Deep Residual Network (ResNet-50) [14], and DenseNet-201 [15]. The efficiency of the proposed concatenated deep learning technique was evaluated using four separate classification networks, when considering industrial production-friendly response (inference) times and accuracies. To the best of our knowledge, this is the first time a deep learning methodology has been used to automatically inspect HDD slider serial numbers.

## 2. Review of CNN Networks

**Figure 2:**
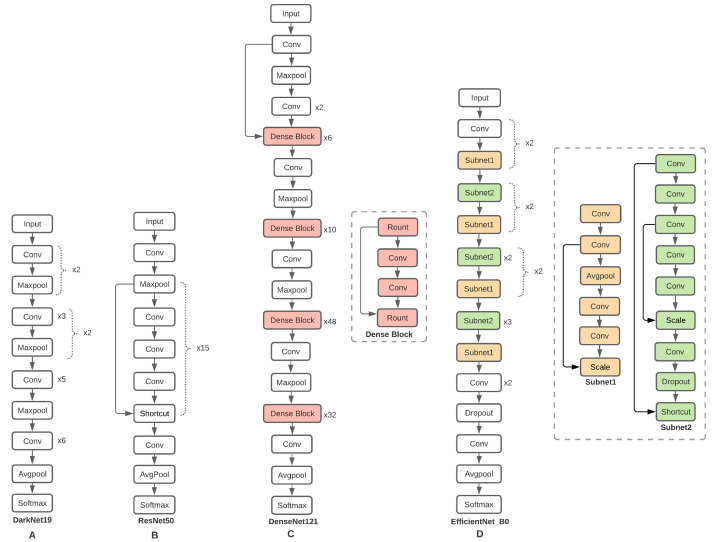
CNN networks used in this study. DarkNet-19 (**A**), ResNet-50 (**B**), DenseNet-121 (**C**), and EfficientNet-B0 (**D**).

CNNs have emerged as the dominant machine learning technique for detecting virtual objects. Since the original Lenet-5 [16], VGG-19 [17], and Highway Networks [18], advances in computer hardware, the availability of large amounts of training data, and advancements in CNN network structure have allowed for increasingly deep training. When the network depth increased, which resulted in a higher training error, a degradation problem arose, in which accuracy became saturated and then quickly degraded. Residual networks (ResNets) [14] are feedforward neural networks with shortcut connections that perform identity mapping by adding their outputs to the outputs of the stacked layers to solve the degradation problem. ResNets are simple to optimize and achieve better accuracy, so plain networks have more training errors as network depth increases. As candidates for classification training, we used ResNets-50 (50 convolutional layers, 1 maxpooling, 1 averagepool, and 16 shortcuts), which produced 76.0% top-1 accuracy and 93.0% top-5 accuracy on ImageNet validation (Figure 2A).

Deep CNN Darknet-19 [12] was proposed as the classification model Darknet-19, which has 19 convolutional layers and 5 maxpooling layers (Figure 2B) and needs only 5.58 billion operations to process an image, while achieving 72.9% top-1 accuracy and 91.2% top-5 accuracy on ImageNet validation. Darknet-19 is less sophisticated, so it is faster and more precise. CNNs with narrower connections between layers close to the output can be significantly deeper, more precise, and more effective to train. The Dense Convolutional Network (DenseNet) [15] connects each layer to every other layer in a feedforward manner (Figure 2C) to enhance the vanishing gradient problem, increase feature propagation, facilitate feature reuse, and decrease the number of parameters significantly. We used DenseNet-201 in this study, which has 179 convolutional layers, 4 maxpools, and 1 averagepool with 20 million parameters. EfficientNet-B0 [13] is a new scaling approach that evenly scales all depth/width/resolution dimensions (Figure 2D). We chose EfficientNet-B0 for image classification training because it has just 5.3 million parameters and 0.39 billion FLOPs, while Resnet-50 has 26 million parameters and 4.1 billion FLOPs. EfficientNet-B0 had a top-1 accuracy of 77.1% and a top-5 accuracy of 93.3%.

## 3. The Concatenated Deep Learning Model

**Figure 3:**
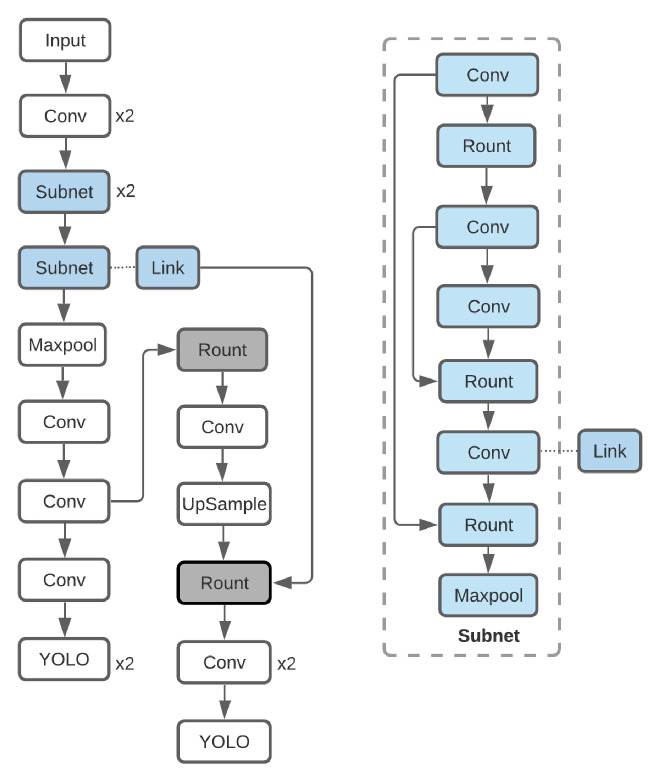
YOLO v4 used for object detection.

**Figure 4:**
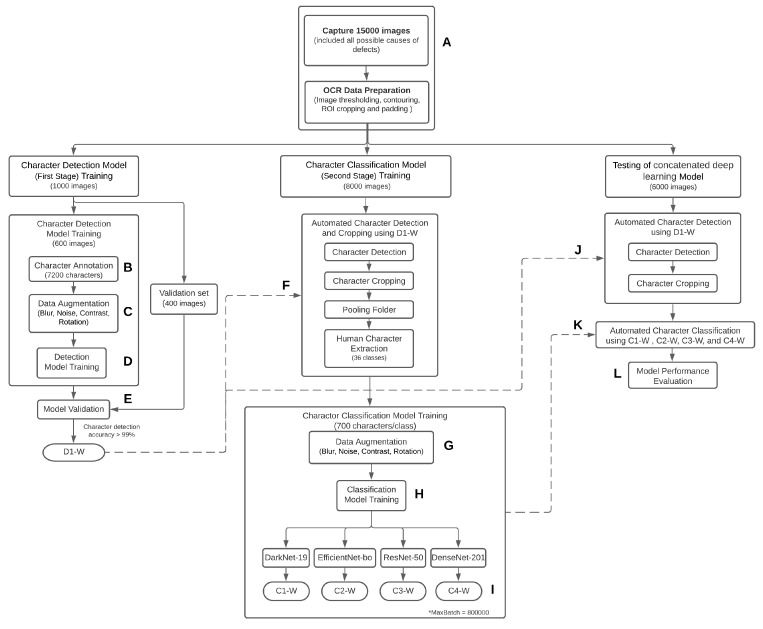
Schematic diagram of the concatenated deep learning model.

The concatenated approach is proposed as a novel technique that consists of two concatenated deep learning models, one for character detection and another for character classification. The excellent performance of the hybrid algorithms for developing an object detection and classification approach for classifying species and gender mosquito vectors [19] was previously published. Furthermore, an approach that combines an object detection model based on an object detection model with one of four classification models, namely Darknet, Darknet19, Darknet19-448, and Densenet-201, was effectively used to characterize the P. gallinaceum avian malaria blood stages [20]. In this work, the character detections for the first stage model were implemented on captured images using the object detection-based You-Only-Look-Once (YOLO) v4 CNN [21] (See Figure 3). Cropped rectangular image regions that were precisely fitted to each observed character served as input datasets for the second stage CNN model. In the second stage, the cropped character images from the first stage were used as the training dataset for the character classification model. The character classification models using four CNN networks were compared: DarkNet-19 [12], EfficientNet-B0 [13], ResNet-50 [14], and DenseNet-201 [15]. Figure 4 illustrates the process flow for the proposed method’s data preparation and model training. The workflow is divided into three sections: image preprocessing, character detection, and character classification.

### 3.1. Captured Image Preprocessing

**Figure 5:**
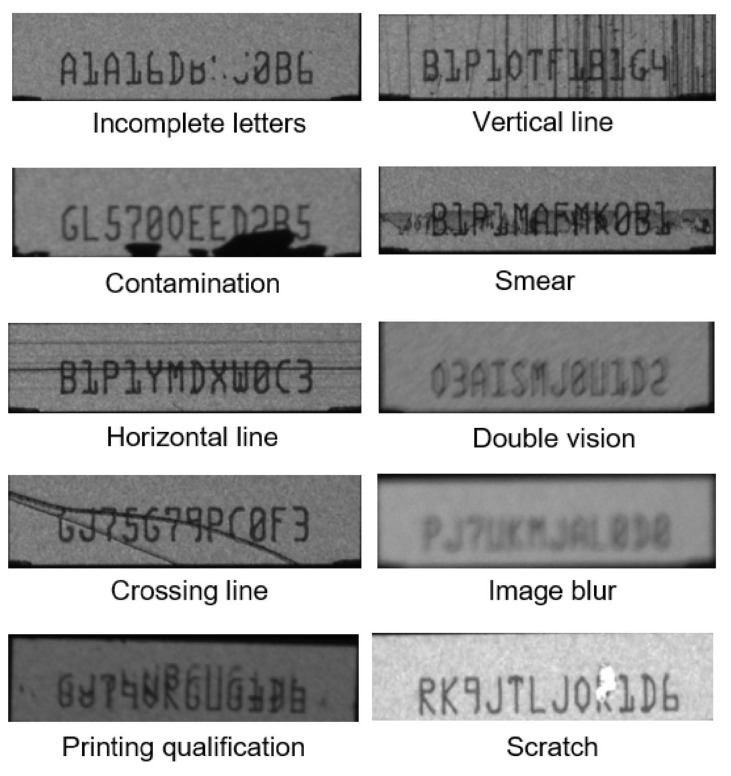
Types of defects on serial number images occurring in a production line.

The defect images (15,000 images) of a slider’s serial number are usually rejected by the industrial standard OCR protocol. Typically, such images were taken by industrial digital cameras (Point Grey 1/3” CCD; mono; 1288 × 964; 31 fps) from 100 head reader/writer machines (150 captured images/machine). Normally, the defect images were identified manually in order to retrieve all potential good sliders from the rejection. Figure 5 depicts all potential sources of defects in an industrial manufacturing line (label A in Figure 4). The defect images have a resolution of 1280 × 960 pixels. The images were divided into three categories: 9000 for the train dataset (1000 for the character detection layer and 8000 for character classification) and 6000 for model tests (Figure 4).

**Figure 6:**
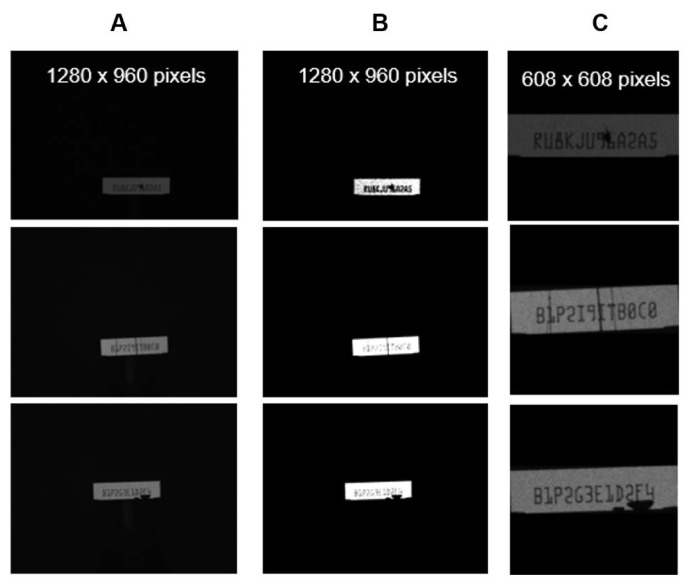
OCR data preparation. (**A**) original captured images with variations of image contrast and shifting of region of interest (ROI), (**B**) thresholding and edge detection, and (**C**) ROI cropping and padding.

During image capture procedures in a typical industrial environment, there are two possible variants that are uncontrollable in the experiment setting (Figure 6A). First, variations in the positioning of the serial number on the captured images are caused by differences in the camera settings and installation of each unit. Second, differences in illumination in various settings culminated in variations in contrast in the captured images. To eliminate such variants, the standard image processing method based on opencv-python 4.4.0.44 [22] was used to extract only the serial number regions and contrast equalization. The Otsu approach [23] was used for image thresholding to generate a binary image (Figure 6B). The contour approximation technique was used to describe the boundaries of the area of interest (ROI), i.e., the serial number background. To keep all images the same size, ROI cropping was used, followed by additional padding on the background. Finally, as seen in Figure 6C, serial number regions were placed in the image’s middle, and the images were sized so that they all had the same height (608 pixels) and width (608 pixels).

### 3.2. The First Stage Model: The Character Detection Model

#### Dataset Preparation and Training

**Figure 7:**
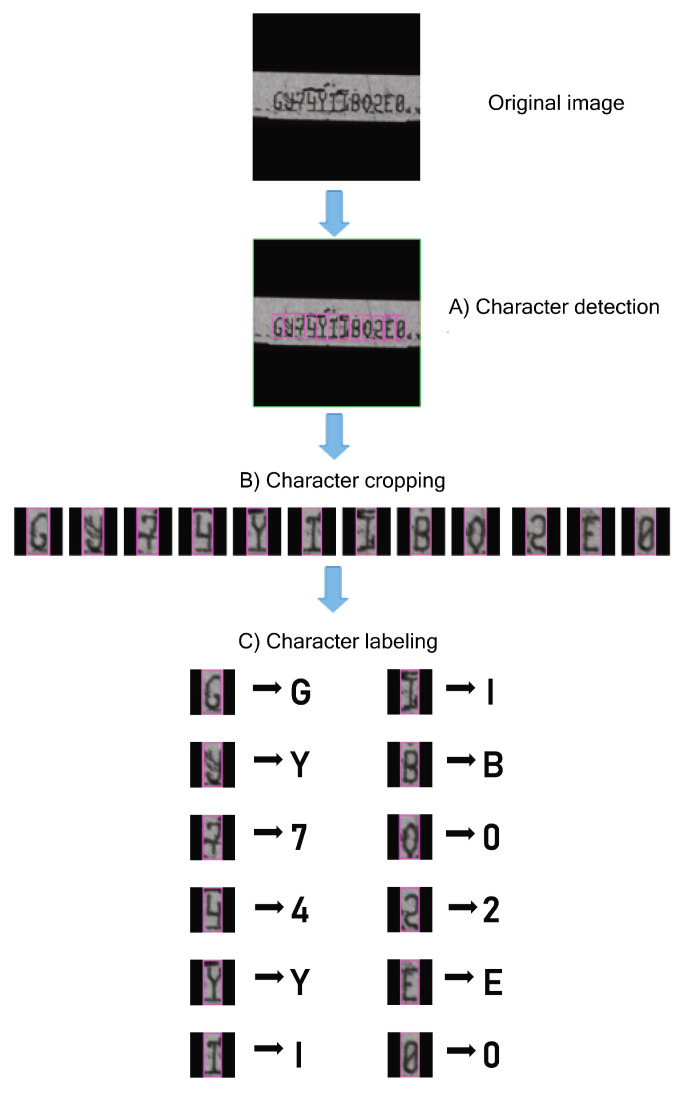
Example of character detection, cropping, and labeling process.

The purpose of this procedure is to create an object detection model for automatically detecting character regions on serial number images obtained from the preprocessed images described previously. To begin, the dataset was prepared through character annotation and augmentation. A dataset of 600 images was chosen at random from the first group of datasets (label B in Figure 4). Character annotation was performed by trained professionals, resulting in 7200 designated character images. We used a bounding box to annotate each character (A–Z and 1–9 classes) on the image (12 characters/image) (35 × 75 pixels). The bounding box’s central point was situated in the center of each character and included the character’s area. Data augmentation was carried out by considering several possible variations (i.e., rotation, blurring, contrast, and noise) from the original images (label C in Figure 4). We rotated the original images from −180° to 180° (90° per step); altered the Gaussian blur filters, which are values of standard deviation of 3, 6, 9, 12, 15, 18, 21, 24, 27, and 30; adjusted the contrast by multiplying all pixel values with 0.8, 1.0, 1.2, 1.4, 1.6, 1.8, 2.0, and 2.2; and increased the Gaussian noise distribution with a standard deviation of 10. Finally, 1,728,000 augmented characters were created. Furthermore, the augmented characters were imported into an in-house deep learning model development platform (CiRA CORE, https://www.facebook.com/groups/cira.core.comm, (accessed on 9 September 2021).to train the character-detection-based CNN model (YOLO v4) (labeled D in Figure 4). For training, a CiRA CORE server with an Intel Xeon^®^silver 4210 CPU2@.2GHzx40 and 125.6 GB RAM was used. The total computation time was 36 h, 15 min, and 52 s. The parameters denoted as D1-weighted (D1-W) were obtained after the model training by choosing the best performance of a character detection model. Finally, the character detection model was tested with D1-weighted with validation data containing 400 images (label E in Figure 4). The resulting detection rate reached 100%, and the detected area of each character can cover the entire bounding box (Figure 7A).

### 3.3. The Second Stage Model: The Character Classification Model

#### 3.3.1. Dataset Preparation

The second group of datasets obtained from 8000 preprocessed defect images was used to develop a character classification model. The character detection model described in Section 3.2 was employed with the loaded optimized D1-weighted parameter. A dataset of 96,000 characters was obtained by performing a cropping operation on the detected character image region (Figure 7B). After that, the cropped character images were manually labeled (Figure 7C) and sorted into 36 classes (0–9 number classes and A–Z alphabet classes). Each class had a total of 700 characters. The dataset was endorsed by two highly experienced observers (label F in Figure 4). There were a total of 25,200 characters used for training of the classification model in the next stage. Following this, data augmentation was carried out by taking into account several possible variants from the original images (label G in Figure 4). To achieve a 630,000-character dataset (17,500 per class), we altered the Gaussian blur filters by varying the values of standard deviation of 3, 6, 9, 12, and 15, adjusting the contrast by multiplying all pixel values with 1.0, 1.3, 1.6, 1.9, and 2.1.

#### 3.3.2. Model Training

For classification model training, the augmented training dataset of 36 classes of 630,000 characters was imported into the in-house deep learning framework (CiRA CORE platform) (label H in Figure 4). Four CNN networks (i.e., DarkNet-19, EfficientNet-B0, ResNet-50, and DenseNet-201) were chosen for performance evaluation using the same dataset. The training parameters were a batch size of 64, momentum of 0.9, decay of 0.0005, maximum batches of 800,000, and learning rate of 0.1 (except for EfficientNet-B0, with a learning rate 0.256). The CiRA CORE server (Intel Xeon^®^silver 4210 CPU2@.2GHzx40, 125.6 GB RAM) was used for performing the training of the character classification model. Table 1 summarizes the training time of four CNN networks on the same training dataset, as well as the parameter settings and computing hardware. The four model parameters C1-W, C2-W, C3-W, and C4-W obtained from the DarkNet-19, EfficientNet-B0, ResNet-50, and DenseNet-201 models, respectively, were used in the performance assessment during the model testing process (label I in Figure 4).

### 3.4. The Model Performance Evaluation

Experiments were carried out to evaluate the performance of the proposed method for automated character identification and serial number classification from defect images. The proposed model was employed to test 6000 defect images for automatic character recognition and cropping using the D1-weighted model (label J in Figure 4). Following this, the four candidate qualified weights (C1-W, C2-W, C3-W, and C4-W) for each model were used for automatic character classification (label K in Figure 4). The experiments were carried out on a server with an Intel Xeon^®^silver 4210 CPU2@.2GHzx40 and 125.6 GB RAM. The test dataset is available at https://github.com/Chousak/OCR-Data-Validation-Noise-.git, (accessed on 25 August 2021).

To measure the classification performance (label L in Figure 4), the performance indexes of Precision, Recall, and Accuracy were calculated:

(1) $$\begin{array}{cc} \mathrm{Precision} & =\frac{\mathrm{TP}}{\mathrm{TP}+\mathrm{FP}} \end{array}$$

(2) $$\begin{array}{cc} \mathrm{Recall} & =\frac{\mathrm{TP}}{\mathrm{TP}+\mathrm{FN}} \end{array}$$

where TP is true positive, FP is false positive, and FN is false negative. This can be defined as accuracy, as shown below, by considering both precision and recall:

(3) $$\begin{array}{cc} \mathrm{Accuracy} & =\frac{\mathrm{TP}+\mathrm{TN}}{\mathrm{TP}+\mathrm{FP}+\mathrm{TN}+\mathrm{FN}} \end{array}$$

where TN is true negative. Accuracy is the best model performance parameter indicator according to the HDD manufacturing perspective, but performance evaluation for imbalanced data, as in this study, can be evaluated more effectively when using the F1 score. The F1 score is a harmonic average of precision and recall rate and can be defined as follows:

(4) $$\begin{array}{cc} F1\;\mathrm{score} & =\frac{2*\mathrm{Precision}*\mathrm{Recall}}{\mathrm{Precision}+\mathrm{Recall}} \end{array}$$

It is important to have at least 99% Accuracy, Precision, Recall, and F1 score for implementation in real-time classification on a production line. To assess the feasibility of the proposed method, the effective inference time of the proposed method should be <300 milliseconds (ms), which is the inference time of the OCR-based computer vision. Inference time (per image) was compared in four different CNN networks, i.e., DarkNet-19, EfficientNet-B0, ResNet-50, and DenseNet-201.

## 4. Performance Evaluation Results

Table 2 displays the performance evaluation metrics for the four candidate CNN versions. Table 2 shows that with the same constraints, all of the proposed methodologies yield the best performance, with accuracies, precision, recalls, and F1 scores greater than 99%. All four CNN models attained greater than 99.90% accuracy (ranging from 99.98% to 99.99%), with EfficientNet-B0 earning the highest F1 score (99.96%).

**Figure 8:**
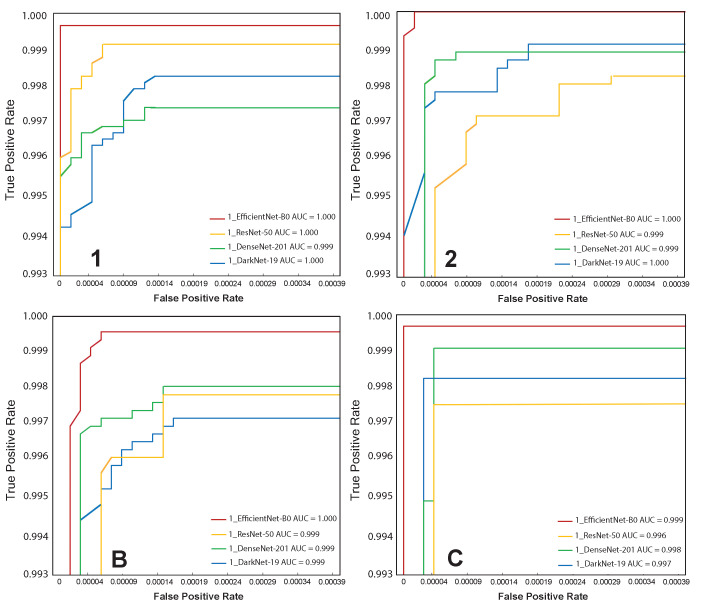
Magnified ROC curves of the top 4 classes from the 4 CNN networks.

**Figure 9:**
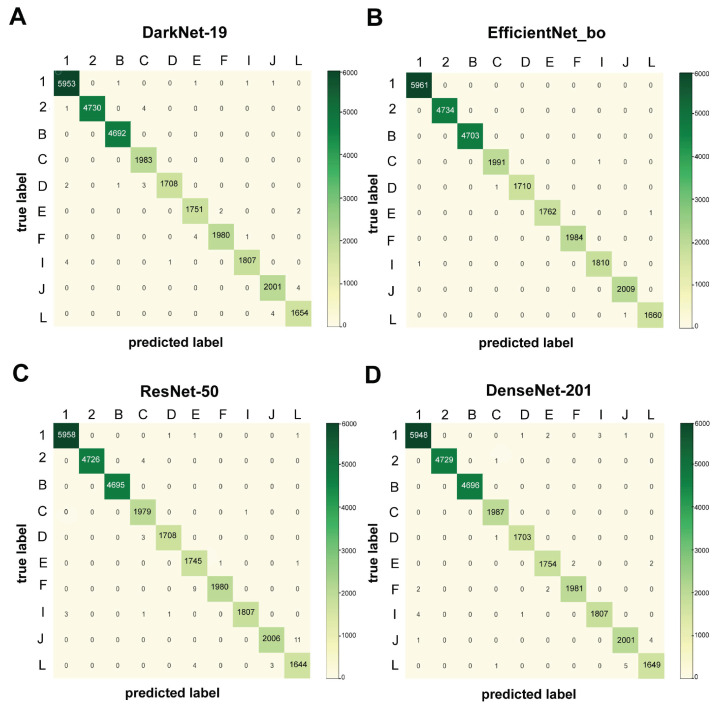
Confusion matrix of character classification of top 10 classes from 4 CNN networks: DarkNet-19 (**A**), EfficientNet-B0 (**B**), ResNet-50 (**C**), and DenseNet-201 (**D**).

Receiver Operating Characteristic (ROC) assessments were also conducted to evaluate the proposed method’s accuracy in the top four groups (i.e., 1, 2, B, and C), which are the characters considered to have the greatest detection errors in the manufacturing process (Figure 8). According to Figure 8, the region under curve (AUC) values for all CNN models is greater than 0.99 in all four classes. As opposed to other CNN versions, EfficientNet-B0 has the highest AUC values (ranging from 0.999 to 1.000) in all four groups (see red lines in Figure 8). Identification errors from 36 classes were counted and sorted to assess the models’ classification performance. To generate confusion matrices, the top 10 groups (i.e., 1, 2, B, C, D, E, F, I, J, and L) with the largest recognition failures were chosen. Figure 9 illustrates the confusion matrices for each model. The color scales reflect the number of projected characters in each class that were compared in four CNN models. The EfficientNet-B0 model was found to have the best classification efficiency. We discovered the following general shortcomings in recognition: ResNet-50 (11 missing), DenseNet-121 (4 missing), and DarkNet-19 (4 missing), with the character “J” misspelled as “L” many times, while EfficientNet-B0 had none. Although the EfficientNet-B0 model produced the best performance, it still takes the most training time (more than 133 h) and is approximately 2.66 times more expensive than the DarkNet-19 model (more than 49 h) (Table 1).

## 5. Discussion

### 5.1. Implementation in Industrial Production

We implemented the system by using the concept of an application program interface (API) service call via the cloud infrastructure to facilitate in the recovery of defects from a standard OCR-based computer vision system in order to minimize computing hardware costs to assist the production lines. To incorporate the proposed system for automatic serial number classification from defect images in production lines, operating costs such as response time (i.e., the amount of inference and communication time) and high-performance computer hardware must be considered. However, the communication time between a typical OCR-based computer vision system and the server can have a limited influence on response time. Nonetheless, the proposed approach can eventually replace traditional OCR-based computer vision when hardware costs are reduced, and the investment is worthwhile. In most OCR processes, checksum measurement is used to ensure that the reading is accurate. In this study, a checksum calculation is employed to verify the results of the proposed model. We can determine the serial number’s checksum as follows:

(5) Checksum=((8∗Checksum)+(ASCIIvalue−32))%59

Serial numbers (12 characters) predicted by the proposed method must be checked. When the checksum does not equal zero, an reading error is indicated. Table 3 shows a checksum calculation example.

To evaluate the benefit of the proposed approach for identifying defective serial number images, we obtained 5000 images from 100 head reader/writer machines on the production line (50 captured images/machine). The recovery rate was estimated as follows:

(6) $$\begin{array}{cc} \mathrm{Recovery}\;\mathrm{rate} & =\frac{\mathrm{Number}\;\mathrm{of}\;\mathrm{identifiable}\;\mathrm{images}}{\mathrm{Total}\;\mathrm{test}\;\mathrm{images}}\;\times \;100\% \end{array}$$

**Figure 10:**
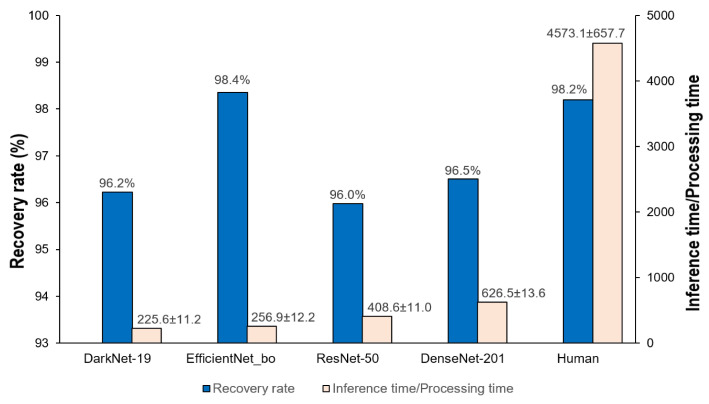
Recovery rates (%) and inference time (second) of four CNN networks and human reading.

**Figure 11:**
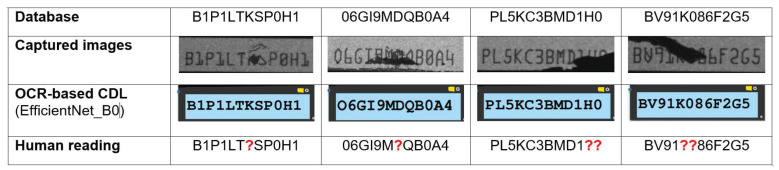
Examples of better classification performances of EfficientNet-B0 superior to human reading.

The overall number of images captured included both identifiable and unidentifiable images. The recovery rates of the four candidate CNN models and human reading are compared to inference time as shown in Figure 10. Darknet-19 and EfficientNet-80 have comparable average inference times of less than 300 ms. As seen in Figure 10, in terms of inference time, all models outperform human operators. However, if we prioritize the recovery rate, the EfficientNet-B0 (98.4%) model outperformed the human reading (98.2%) with faster inference times. Surprisingly, the EfficientNet-B0 model can distinguish those defective serial number images that humans were unable to inspect. Figure 11 shows four examples of serial number images that cannot be recognized by humans. According to Figure 11, the EfficientNet-B0 correctly recognized all four serial number images (checksum result = 0).

To ensure the generality of our model, five-fold cross validation was performed to validate if our chosen model consistently provided general accuracy with no prediction bias [24,25]. Cross-validation, according to our findings, eliminates the problem of overfitting. This is due to the fact that cross-validation can help minimize cross-validated errors in order to create the best model, resulting in statistical parameters that are indifferent between any experiment (Table 4). Furthermore, in order to confirm the robustness of the proposed model, we evaluated it on the serial number dataset with added adversarial noise [26]. The produced adversarial noise’s Adversarial Noise Scale (ANS) parameter was adjusted from 0.01 to 0.5. The performance of the proposed model with various ANS settings is shown in Table 5. The suggested model’s performance was relatively maintained at the same levels with no adversarial noise until the ANS value was more than 0.05. This implies that additional development is required to reduce the influence of adversarial noise in the future model. In order to demonstrate the suggested model’s applicability for tasks other than HDD serial numbers, we evaluated it with a dataset of bank note serial numbers [27]. Table S1 shows the performance of the proposed model using the bank note serial number dataset. The results demonstrate that our proposed model performed at an outstanding level, which is consistent with the results obtained using the HDD serial number dataset.

### 5.2. Limitations of the Proposed Model

Future research should focus on improving the proposed process. First, the proposed method’s generalizability is still minimal. Despite the fact that the proposed model was trained using dataset images that included all practicable and uncontrollable variants in the real industrial context, the proposed model was only trained on a single font type. When the font styles of the serial numbers differ and vary from those of the training dataset, misrecognized font issues can be exacerbated. To boost the generalization of the model, model adaptation should be done by using other font forms of serial numbers in the training process. Nonetheless, misrecognized fonts occurred in the standard OCR process [18]. Furthermore, as new features emerged on images, the OCR data preparation process using image processing methods (image thresholding, contouring, ROI cropping, and padding) could not be done. As a difficult model to develop in a future study, we propose a three-stage deep learning model, which uses a deep learning approach to detect ROI areas in the first layer rather than the aforementioned image processing approaches.

## 6. Conclusions

In this paper, we introduce a concatenated method for character recognition to reduce serial number identification failures on hard disk drive head sliders from the traditional OCR-system-based computer vision. On all four CNN networks, the proposed model offered precision greater than 99%; furthermore, the EfficientNet-B0 network had the highest recovery rate, which was preferable to human reading. The inference times of DarkNet-19 and EfficientNet-B0 were less than 300 ms, which was more than 15 times faster than the inference time of a human reading. As a result, Our proposed method outperformed human reading in character classification. When hardware costs are minimized and the investment is worthwhile, the proposed solution could potentially replace conventional OCR-based computer vision.

## Figures and Tables

**Table 1:** Training time of four CNN networks.

CNN Networks		Training Time	
	Hours	Minutes	Seconds
DarkNet-19	49	58	51
EfficientNet-B0	133	13	4
ResNet-50	82	57	45
DenseNet-201	128	3	4

**Table 2:** Performance evaluation metrics of the proposed method compared among four CNN networks.

CNN Networks	Accuracy	Precision	Recall	F1 Score
DarkNet-19	99.98	99.66	99.73	99.69
EfficientNet-B0	99.99	99.96	99.96	99.96
ResNet-50	99.98	99.61	99.71	99.66
DenseNet-201	99.98	99.70	99.75	99.72

**Table 3:** Checksum calculation of the serial number “FJ72Q4A9P2F4”.

Serial	ASCII	Checksum Equation	Checksum
F	70	((8 × 0) + (70 − 32)) % 59	38
J	74	((8 × 38) + (74 − 32)) % 59	51
7	55	((8 × 51) + (55 − 32)) % 59	18
2	50	((8 × 18) + (50 − 32)) % 59	44
Q	81	((8 × 44) + (81 − 32)) % 59	47
4	52	((8 × 47) + (52 − 32)) % 59	42
A	65	((8 × 42) + (65 − 32)) % 59	15
9	57	((8 × 15) + (57 − 32)) % 59	27
P	80	((8 × 27) + (80 − 32)) % 59	28
2	50	((8 × 28) + (50 − 32)) % 59	6
F	70	((8 × 6) + (70 − 32)) % 59	27
4	52	((8 × 27) + (52 − 32)) % 59	0
			Final result =“0”

**Table 4:** Five-fold cross validation of the EfficientNet-B0 networks from selected data.

k-Folds	Character	Accuracy	Precision	Recall	F1 Score
	1	1.0000	1.0000	1.0000	1.0000
	2	0.9997	0.9887	1.0000	0.9943
	B	1.0000	1.0000	1.0000	1.0000
	C	0.9998	0.9943	1.0000	0.9972
Fold1	D	1.0000	1.0000	1.0000	1.0000
	E	0.9998	0.9943	1.0000	0.9972
	F	0.9998	1.0000	0.9943	0.9971
	I	1.0000	1.0000	1.0000	1.0000
	J	1.0000	1.0000	1.0000	1.0000
	L	1.0000	1.0000	1.0000	1.0000
	Average	0.9999	0.9983	0.9983	0.9986
	1	1.0000	1.0000	1.0000	1.0000
	2	1.0000	1.0000	1.0000	1.0000
	B	0.9995	0.9943	0.9886	0.9914
	C	0.9997	0.9887	1.0000	0.9943
Fold2	D	0.9997	1.0000	0.9886	0.9943
	E	1.0000	1.0000	1.0000	1.0000
	F	1.0000	1.0000	1.0000	1.0000
	T	0.9998	0.9943	1.0000	0.9972
	J	0.9997	0.9887	1.0000	0.9943
	L	0.9992	0.9775	0.9943	0.9858
	Average	0.9998	0.9972	0.9971	0.9957
	1	0.9998	1.0000	0.9943	0.9971
	2	0.9997	0.9943	0.9943	0.9943
	B	0.9986	0.9716	0.9771	0.9744
	C	0.9995	0.9943	0.9886	0.9914
Fold3	D	0.9997	0.9887	1.0000	0.9943
	E	0.9983	1.0000	0.9371	0.9676
	F	0.9976	0.9348	0.9829	0.9582
	I	0.9995	0.9943	0.9886	0.9914
	J	0.9994	0.9942	0.9829	0.9885
	L	0.9989	0.9941	0.9657	0.9797
	Average	0.9994	0.9903	0.9900	0.9837
	1	0.9997	1.0000	0.9886	0.9943
	2	1.0000	1.0000	1.0000	1.0000
	B	0.9995	0.9943	0.9886	0.9914
	C	0.9994	1.0000	0.9771	0.9884
Fold4	D	0.9998	0.9943	1.0000	0.9972
	E	0.9995	1.0000	0.9829	0.9914
	F	0.9990	0.9669	1.0000	0.9831
	I	0.9994	0.9942	0.9829	0.9885
	J	0.9998	0.9943	1.0000	0.9972
	L	0.9995	0.9831	1.0000	0.9915
	Average	0.9995	0.9918	0.9917	0.9923
	1	0.9992	0.9722	1.0000	0.9859
	2	0.9995	0.9886	0.9943	0.9915
	B	0.9990	0.9942	0.9714	0.9827
	C	0.9997	1.0000	0.9886	0.9943
Fold5	D	0.9989	0.9719	0.9886	0.9802
	E	0.9994	0.9942	0.9829	0.9885
	F	0.9984	0.9609	0.9829	0.9718
	I	0.9995	1.0000	0.9829	0.9914
	J	0.9992	0.9830	0.9886	0.9858
	L	0.9992	0.9885	0.9829	0.9857
	Average	0.9995	0.9901	0.9903	0.9857

**Table 5:** Adversarial noise validation of the EfficientNet-B0 networks from selected data.

ANS	Character	Accuracy	Precision	Recall	F1 Score
	1	0.9995	0.9831	1.0000	0.9915
	2	1.0000	1.0000	1.0000	1.0000
	B	0.9979	1.0000	0.9257	0.9614
	C	0.9997	0.9887	1.0000	0.9943
ANS = 0.01	D	0.9994	0.9886	0.9886	0.9886
	E	0.9981	1.0000	0.9314	0.9645
	F	0.9989	0.9773	0.9829	0.9801
	I	0.9990	0.9829	0.9829	0.9829
	J	0.9990	0.9669	1.0000	0.9831
	L	0.9986	0.9770	0.9714	0.9742
	Average	0.9990	0.9864	0.9783	0.9821
	1	0.9994	0.9886	0.9886	0.9886
	2	0.9994	0.9831	0.9943	0.9886
	B	0.9994	0.9886	0.9886	0.9886
	C	0.9997	0.9887	1.0000	0.9943
ANS = 0.02	D	0.9995	1.0000	0.9829	0.9914
	E	0.9979	0.9709	0.9543	0.9625
	F	0.9982	0.9713	0.9657	0.9685
	I	0.9992	0.9885	0.9829	0.9857
	J	0.9994	0.9886	0.9886	0.9886
	L	0.9987	0.9613	0.9943	0.9775
	Average	0.9991	0.9829	0.9840	0.9834
	1	0.9983	0.9940	0.9486	0.9708
	2	0.9971	0.9389	0.9657	0.9521
	B	0.9964	0.9753	0.9029	0.9377
	C	0.9951	0.9011	0.9371	0.9188
ANS = 0.03	D	0.9968	1.0000	0.8914	0.9426
	E	0.9914	0.8735	0.8286	0.8504
	F	0.9844	0.6577	0.9771	0.7862
	I	0.9949	0.9801	0.8457	0.9080
	J	0.9905	0.7576	1.0000	0.8621
	L	0.9887	0.7368	0.9600	0.8337
	Average	0.9934	0.8815	0.9257	0.8962
	1	0.9944	0.9805	0.8629	0.9179
	2	0.9911	0.8626	0.8971	0.8796
	B	0.9859	0.9649	0.6286	0.7612
	C	0.9802	0.6990	0.7829	0.7385
ANS = 0.04	D	0.9897	1.0000	0.7143	0.8333
	E	0.9723	0.6484	0.4743	0.5479
	F	0.9538	0.4233	0.9143	0.5787
	I	0.9845	0.9626	0.5886	0.7305
	J	0.9210	0.2969	0.9943	0.4573
	L	0.9640	0.4923	0.9143	0.6400
	Average	0.9737	0.7331	0.7771	0.7085
	1	0.9861	0.9840	0.7029	0.8200
	2	0.9861	0.8623	0.8229	0.8421
	B	0.9701	0.9524	0.3429	0.5042
	C	0.9514	0.4598	0.6857	0.5505
ANS = 0.05	D	0.9786	1.0000	0.5200	0.6842
	E	0.9607	0.6047	0.2971	0.3985
	F	0.8885	0.2424	0.8229	0.3745
	I	0.9743	0.9405	0.4514	0.6100
	J	0.8735	0.2390	0.9943	0.3854
	L	0.9259	0.3290	0.7257	0.4528
	Average	0.9495	0.6614	0.6366	0.5622

## Data Availability

The data presented in this study are available on request from the corresponding author. The data are not publicly available due to the request of the company that provided the data.

## Supplementary Materials

The following are available online at https://www.mdpi.com/article/10.3390/s21186261/s1, Table S1: Bank note data validation.
