# Supplementary material for: A Deep Learning System for Recognizing and Recovering Contaminated Slider Serial Numbers in Hard Disk Manufacturing Processes
Source: Sensors (Basel). 2021 Sep 18;21(18):6261. doi: 10.3390/s21186261 (PMC8472306; doi:10.3390/s21186261)
Supplement: Supplementary file 1 [file sensors-21-06261-s001.zip › sensors-1334527-supplementary.pdf]

Table S 1: Bank note data validation.

Character	Accuracy	Precision	Recall	F1 score
0	0.9996	0.9987	0.9962	0.9974
1	0.9999	1.0000	0.9993	0.9996
2	0.9998	0.9987	0.9996	0.9991
3	0.9999	1.0000	0.9996	0.9998
4	0.9999	0.9995	1.0000	0.9997
5	0.9999	1.0000	0.9994	0.9997
6	0.9998	1.0000	0.9978	0.9989
7	0.9999	0.9995	0.9995	0.9995
8	1.0000	1.0000	1.0000	1.0000
9	1.0000	1.0000	1.0000	1.0000
A	0.9999	1.0000	0.9989	0.9994
B	0.9976	1.0000	0.9400	0.9691
C	0.9996	0.9858	0.9984	0.9921
D	0.9999	1.0000	0.9984	0.9992
E	0.9976	0.9631	0.9631	0.9631
F	0.9982	0.9404	0.9693	0.9546
H	1.0000	1.0000	1.0000	1.0000
J	0.9999	0.9943	1.0000	0.9971
K	1.0000	1.0000	1.0000	1.0000
L	0.9999	0.9979	0.9959	0.9969
N	1.0000	1.0000	1.0000	1.0000
P	1.0000	1.0000	1.0000	1.0000
R	0.9982	0.6626	1.0000	0.7971
S	0.9999	0.9859	1.0000	0.9929
T	1.0000	1.0000	1.0000	1.0000
U	1.0000	1.0000	1.0000	1.0000
V	1.0000	1.0000	1.0000	1.0000
W	1.0000	1.0000	1.0000	1.0000
X	0.9999	0.9230	1.0000	0.9600
Y	1.0000	1.0000	1.0000	1.0000
Z	1.0000	1.0000	1.0000	1.0000
Average	0.99968	0.98226	0.99536	0.98761
